# Drug Safety Monitoring in Health Programs of Cameroon

**DOI:** 10.35248/2329-6887.22.10.385

**Published:** 2022-10-17

**Authors:** Ketina Hirma Tchio-Nighie, Maurice Mbwe Mpoh, Herve Tchokomeni, Ingrid Marcelle Koutio Douanla, Paul Nyibio Ntsekendio, Frank Forex Kiadjieu Dieumo, Jerome Ateudjieu

**Affiliations:** 1 Department of Health Research, M.A. SANTE (Meilleur Acces auxSoins de Sante), Yaounde, Cameroon; 2 Department of Pharmacy, Drugs and Laboratories, Ministry of Public Health of Cameroon, Yaounde, Cameroon; 3 Department of Drugs and Population’s Health, Bordeaux, France; 4 Department of Public Health, University of Dschang, Dschang, Cameroon; 5 Department of Statistics, Sub-Regional Institute of Statistics and Applied Economics, Yaounde, Cameroon; 6 Department of Economics and Management, University of Yaounde I, Yaounde, Cameroon; 7 Department of Health Operations Research, Ministry of Public Health of Cameroon, Yaounde, Cameroon

**Keywords:** Drug safety monitoring, Drug safety, Clinical trials, Health programs

## Abstract

**Background:**

Data are needed to serve as evidence in planning the strengthening of pharmacovigilance in health programs administering drugs to populations. The present study was proposed to map the distribution of pharmacovigilance units in health programs, assess the availability of key resources, the implementation of key pharmacovigilance activities and identify needs of involved actors.

**Methods:**

It was a cross sectional descriptive study targeting all health programs of the Cameroon Ministry of Public Health administering drugs/vaccines to the population. Data were collected using semi structured questionnaire administered face to face to key persons in charge of drug safety monitoring or drug management in health programs.

**Results:**

Out of the 09 health programs involved in drug distribution, 07 consented to participate. Five out of them (71.4%) claimed to have existing pharmacovigilance units. Office space, computers, operating budget, data analysis software and a trained staff were available in 28.6%, 42.9%, 42.9%, 14.3%, 00.0%, and 42.9% of the health programs respectively. One of the 7 health programs (14.3%) declared conducting detection/notification of adverse events following exposure drugs, 2 (28.6%) conduct causality assessment and 3 (42.8%) conduct analysis of pharmacovigilance data. All health programs proposed to prioritize the allocation of budget and qualified personnel and the training of existing personnel as key interventions to improve drugs/vaccines safety monitoring in health programs.

**Conclusion:**

The study reports limited coverage of Cameroon health programs with activities leading to drugs and vaccine safety monitoring. Suggested actions have to be taken into account when attempting to improve the situation.

## Introduction

Information on drug safety at the end of clinical trials is limited to guide the updating of risk benefit ratio of marketed drugs and vaccines. This is due to the fact that participants selection during clinical trials is more rigorous than when drug is administered to patients in real life, the sample sizes and the duration of follow up of clinical trials participant are limited to allow the detection of rare and chronic adverse events following exposure to drugs [1–3]. In addition, the appropriateness of the design of some clinical trial in detecting adverse events has been questioned by some studies [4]. This is illustrated by differences in the distribution of reported adverse events before and after the clinical trials [5,6]. In response to these weaknesses, WHO recommends in each country and in organizations supplying drugs to population to put in place a system to permanently ensure the monitoring of drug safety [7,8]. This is part of Cameroon Ministry of Public Health (CMOPH) policy and implemented by creating in the ministry of public health a unit to organize and coordinate drug safety monitoring [9].

Studies conducted in many settings underline under reporting and limited resources as limitations in the planning and implementation of drug safety monitoring activities [10,11]. Many interventions have been implemented to improve reporting and investigation rate of Adverse Events Following Exposure to Drugs or Vaccine (AEFED) [12–14]. The implementation of recommendations resulting from these studies need the assessment of the organization of drug safety monitoring.

Due to the burden of health problems in Cameroon, health authorities have put in place health programs that are in charge of implementing interventions to improve population access to care. Despite the fact that it is not described by any study, it is of common knowledge that most health programs are involved in acquisition and distribution of drugs. As indicated in the related guidelines, drug safety monitoring has to be part of health programs involved in drugs’ supply and use [15]. To the best of our knowledge, no study has been conducted in Cameroon describing the existence, performance and organization of drug safety monitoring in health programs. Such study is expected to provide evidence that can be used to identify needs of interventions to ensure protection of populations that are exposed to drugs distributed by health programs. The present study is proposed to map the distribution of pharmacovigilance units in health programs, assess the availability of key resources, describe the implementation of key pharmacovigilance activities and identify perceived needs of the authorities involved in drugs management or safety monitoring in health programs.

## Methodology

### Study design

It was a cross sectional descriptive study targeting all Health Programs of the Cameroon Ministry of Public Health (CMOPH) involved in drug acquisition and distribution to the population. Data were collected by trained surveyors using a questionnaire administered face to face to key persons in charge of drug safety monitoring or drug management in health programs.

### Study area and period

The study was carried out in headquarters of health programs based in CMOPH head offices in Yaoundé. Data were collected from October to December 2020.

### Study population

All health programs involved in acquisition and supply of drugs to population were eligible. The respondents had to be chosen by the program coordinator and had to be involved in the organization of drug safety monitoring. Health programs that refused to participate in the study were excluded.

### Data collection tool and variables

The data collection form was developed by the study team and administered as pretested to two key persons familiarized with drug management in Cameroon health programs. Key variables included in this form were the involvement of the health program in drug distribution, the existence of pharmacovigilance units, the availability of infrastructures and key resources, pharmacovigilance key activities, the knowledge of key persons regarding these activities and their perceived needs regarding pharmacovigilance.

### Study implementation procedure

In each of the health programs, the head of the program was met and was presented study procedures, objectives and briefed on who is eligible to respond to the study questionnaire. If consenting, the head of the program had to indicate the eligible respondent. The proposed person was met to be informed before deciding to participate. Those consenting to participate were administered a face-to-face questionnaire.

### Data management and analysis

Data were collected using an ODK (Open Data Kit) interface and uploaded online. The online data base was downloaded at daily bases and processed in order to identify and correct errors and inconsistencies. During the data collection period, daily meetings were held between the data management team and field data collection supervisors to solve detected data problems.

Data were analyzed using Epi Info software by estimating proportion of health programs with existing pharmacovigilance units, availability of infrastructure, equipment and tools; implementation of detection, reporting, investigation, causality analysis, the proportion of respondents mastering the level of implementation of pharmacovigilance activities and their perceived needs.

### Ethical consideration

The present study was conducted expecting to provide evidence to improve the protection of population exposed to drugs delivered by health programs. Key information was communicated to head of health programs and respondents; their written consent was obtained before questionnaire administration. No link connecting collected data to health programs was collected or generated. The protocol was approved by the Cameroon National Ethics committee for research in Human Health. The reference of the ethical clearance is: No.2020/10/1304/CE/CNERSH/SP.

## Results

### Characteristics of health program and respondent’s status

Of 09 targeted health programs, 07(77.8%) consented to participate. The participating health programs include: National Onchocerciasis Control Program (NOCP), National Malaria Control Program (NMCP), Expanded Immunization Program (EIP), National Schistosomiasis Control Program (NSCP), National Tuberculosis Control Program (NTCP), National Buruli Ulcer Control Program (NBUCP) and the National AIDS Control Program (NACP).

The status of respondents in their respective health programs is presented in Table 1. Four (57.1%), two (28.6%) and one (14.3%) were pharmacists, doctors and epidemiologist respectively (Table 1).

### Existence of pharmacovigilance units and pharmacovigilance activities in health programs

Five (71.43%) of the 7 health programs claimed to have a pharmacovigilance unit. Pharmacovigilance activities claimed to be carried are presented in Table 2. Three (42.9%) of the 7 health programs do not organize the detection, reporting, nor causality assessment of adverse event following exposure to drugs or to vaccines.

### Availability of infrastructures, equipment and tools

The distribution of infrastructure, equipment and tools for pharmacovigilance is presented in Table 3. Only 2 (28.6%) out of the included health programs had an office space. Three (42.9%), four (57.1%), and one (14.3%) had a trained staff, a data archiving system and an operating budget for pharmacovigilance activities respectively. No health programs reported to have data analysis software.

### Knowledge on the level of implementation of pharmacovigilance activities

**Figure 1:**
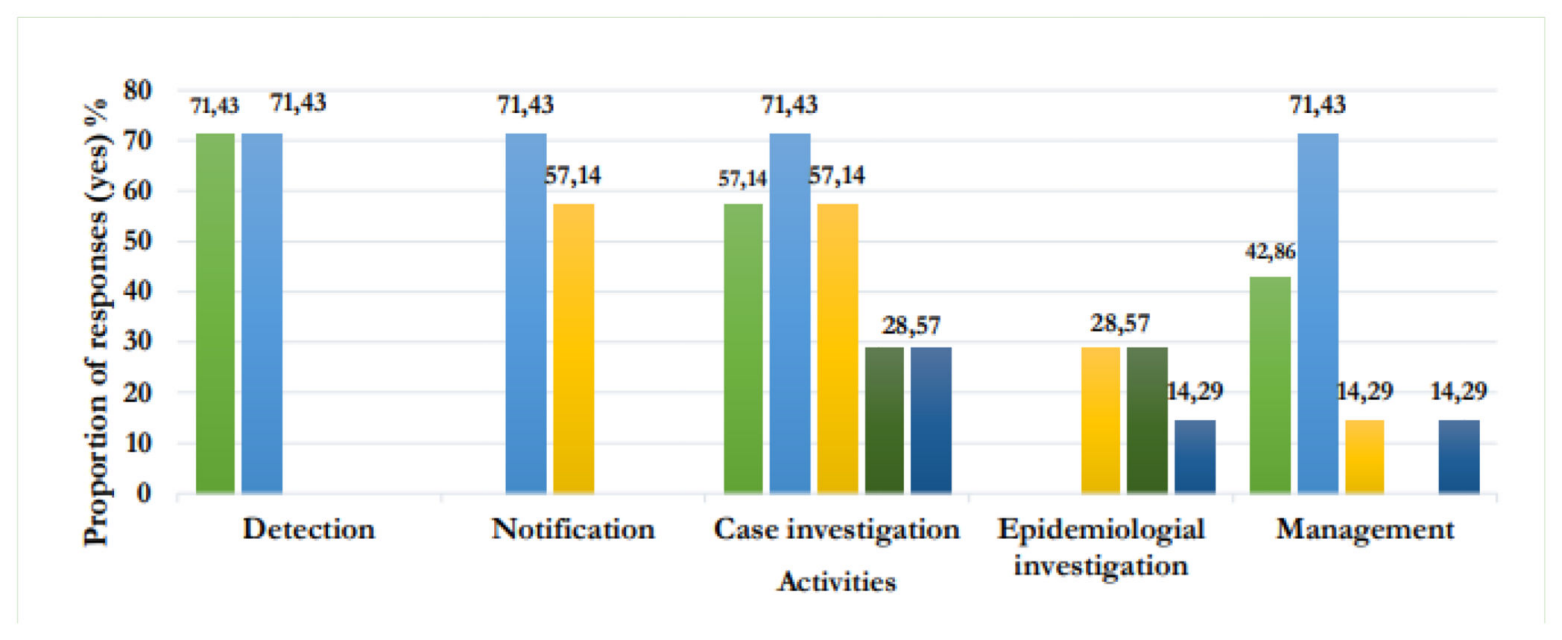
Distribution of respondents’ knowledge on the level of implementation of pharmacovigilance activities. **Note:** (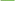) Community; (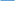) Health facility; (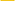) Health district; (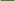) Regional delegation of public health; (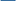) DPML (Department of Pharmacy, Drugs and Laboratories).

Figure 1 presents the distribution of knowledge of respondents regarding the level of implementation of AEFED key activities. In the case of all the activities, not all the respondents knew their appropriate level of implementation.

### Analysis on pharmacovigilance data

Of the 7 health programs, 4 (57.1%) reported not conducting any analysis on pharmacovigilance data. The frequency of data analysis is done annually and weekly in 2 and 1 of the 3 health programs conducting data analysis. A given fraction of health programs involved in data analysis declared to send feedbacks on their data analysis to reporting services. These included 2 on 3 to reporting health facilities, to health districts and to the regional delegation of public health. The completeness of forms, timeliness of the reporting and investigation rates were claimed to be estimated during data analyses by only one of the 3 health programs analyzing their data.

### Perceived needs of health programs respondent to improve drugs safety monitoring

Table 4 presents perceived needs of health programs respondents to improve drug safety monitoring in their programs. The perceived needs of the different health programs are presented in Table 4. All the health programs declared the need for allocation of budget and qualified personnel and the training of existing personnel as key interventions to improve drug safety monitoring.

## Discussion

The present study revealed that the coverage of Cameroon health programs in terms of pharmacovigilance unit was 5 (71.4%) out 7 by the end of 2020. By the same time, out of 7 health programs, office space, computer, trained staff, operating budget, and data analysis software were available in 2 (28.6%), 3 (42.9%), 3 (42.9%), 1 (14.3%), and 0 (00.0%) respectively. One out of 7 (14.3%) Health programs declared conducting detection/reporting and 2 out of 7 (28.6%) conduct causality assessment or AEFED. Four (57.1%) out of 7 health programs declared not to conduct analysis on pharmacovigilance data. Assigning a budget line, organizing the training of staff, hiring qualified human resources, were perceived as needs by health programs.

Targeted health programs are those known to be involved in the acquisition and distribution of drugs to populations including children, pregnant women and men. International and local regulations recommend in this case, the establishment of a monitoring system for drug safety. Information on the implementation of this recommendation is to the best of our knowledge not available in Cameroon context. However, it is essential to guide the decision-makers in charge and to stimulate needed corrective action. In the present study, up to two health programs supplying drugs to the population were not having any unit organizing the monitoring of the safety of these drugs. Even though it is possible to conduct pharmacovigilance activities without a unit, its presence is needed to ensure the planning of activities, mobilize resources, ensure the training and monitoring of involved health personnel and entities. Subject to the fact that this study did not evaluate the performance of the declared existing pharmacovigilance units, we recommend not only to evaluate the performance of these existing units but also to advocate decision makers to ensure that health programs have performant units of pharmacovigilance.

All structures involved in drug supply have to contribute to its safety by participating in the detection, reporting, investigation and causality assessment of adverse drug events following exposure to drugs. The present study indicates that very few health programs fulfil each of these tasks. It could be inferred from these results that data on known and unknown serious adverse events resulting from population exposure to drugs are not available to serve as evidence to perform risk benefit analysis of these drugs for the majority of health programs. This is expected to handicap health systems in catching up with the limitation of clinical trials regarding drug safety monitoring given the fact that previous studies have shown that the distribution of adverse events following exposure to drugs or vaccines can differ between clinical trials and marketed phase of drugs development [16]. Regulation should be put in place by the Cameroon health system to ensure that all structures involved in the supply of medicines include the recommended medicine safety monitoring activities.

Given that pharmacovigilance involves an important number of activities in full time involving many teams for detection, reporting, investigation, supervision and training, there is a need to have an office space, qualified personnel, an operating budget, computers and archiving systems. This study revealed that the majority of health programs did not have the minimum required infrastructure, equipment and tools. These elements should be taken into account when planning resources and activities of health programs. The fact that they are lacking in a number of health programs indicates a real need for action targeting decision-makers to ensure that resources for monitoring drug safety are included in the planning of drug and vaccine supply activities. Similar situations have been described in other settings, underlying the need to include resource supply for pharmacovigilance in international policies supporting health programs.

The present study revealed participants perceived and documented needs regarding pharmacovigilance activities and the high concordance between these needs. This concordance is for greatest importance in the process of planning and monitoring the response to these needs. The content of these needs is consistent with studies conducted in other settings [17,18]. The needs observed in the present study have to be taken into account in the elaboration of strategies for drug safety monitoring.

The present study had some limitations. All the targeted health programs could not be enrolled in the study because some did not consent to participate. We cannot determine if data on the 2 health programs that did not consent to participate in this study could have changed its results. Those who answered the questions were part of the team in charge of implementing pharmacovigilance in the health programs and in this case being in a situation of conflict of interest to disclose weakness of activities they are in charge of. This situation, which could not be avoided in the context of the present study is likely to induce information bias. Despite these limitations, we believe that the study identified limitations to be overcome in order to improve the pharmacovigilance system of health programs in Cameroon.

## Conclusion

The results of the present survey reveal the existence of a weakness in health programs in terms of existence of pharmacovigilance units where only 5 on 7 do have a unit. It also underlined unavailability of key resources like an office space, qualified personnel, an operating budget and appropriate office equipment/tools in majority of health programs. Activities necessary for the monitoring of drug safety were not implemented in most health programs. Most of health programs identified needs of budget line, hiring of trained staff, organization of training and development of regulation and guidelines to improve drugs safety monitoring. From findings of this study, we recommend to the authorities in charge of pharmacovigilance in health programs to advocate decision makers to ensure that health programs involved in drugs supply have units of pharmacovigilance, take into account resources and equipment for pharmacovigilance when planning resources and activities of health programs, take into account the needs perceived and documented needs of health programs respondents by the respondents and define regulations that include the organization of drug safety monitoring in new and old health programs.

## Figures and Tables

**Table 1:** Health programs respondents status

Respondent status	Frequency (N=7)	Percent (%)
Head of pharmacovigilance unit	4	57.1
Pharmacovigilance focal point	2	28.6
Head of cases management unit	1	14.3

**Table 2:** Distribution of pharmacovigilance activities reported to be organized by responding health programs

Activities	Frequency (N=7)	Percent (%)
Detection of Adverse Events Following Exposures to Drugs or Vaccines (AEFED)	1	14.3
Reporting of AEFED	1	14.3
Causality assessment of serious AEFED	2	28.6
No activity	3	42.9

**Table 3:** Availability of infrastructures, equipment and tools for the pharmacovigilance

Modality	Frequency (N=7)	Percent (%)
	**Infrastructure and Equipment**	
Computer	3	42.9
Office space	2	28.6
	Resources	
Trained staff	3	42.9
Operating budget	1	14.3
	Surveillance Tools	
AEFED case report form	6	85.7
Forms archiving system	4	57.1
Case investigation sheet	4	57.1
Pharmacovigilance guide	4	57.1
A telephone line for AEFED surveillance	1	14.3
A reporting website	1	14.3
Data analysis software	0	0

**Table 4:** Perceived needs of health programs to improve drugs safety monitoring

Needs	Frequency (N=7)	Percent (%)
Allocation of budget line for pharmacovigilance	7	100
Training of key staff on pharmacovigilance	7	100
Hiring of qualified human resources to lead activities	7	100
Development of regulatory text organizing pharmacovigilance	3	42.9
Development of adapted pharmacovigilance guidelines	3	42.9
Need to have a team for the investigation of AEFED	2	28.6

## Data Availability

The datasets used and/or analyzed during the current study are available from the corresponding author for reasonable requests.
